# Neural modifications of transtibial prosthesis (TTP) users: an event-related potentials study

**DOI:** 10.1186/s12984-025-01606-y

**Published:** 2025-03-26

**Authors:** Ampika Nanbancha, Weerawat Limroongreungrat, Manunchaya Samala, Jutima Rattanakoch, Gary Guerra, Wisavaporn Niamsang, Kittichai Tharawadeepimuk

**Affiliations:** 1 College of Sports Science and Technology, Mahidol University, Nakhon Pathom, 73170 Thailand; 2 Sirindhorn School of Prosthetics and Orthotics, Faculty of Medicine Siriraj Hospital, Mahidol University, Bangkok, 10700 Thailand; 3 Exercise and Sport Science Department, St. Mary’s University, San Antonio, TX 78228 USA

**Keywords:** Transtibial prosthesis (TTP) users, Event-related potentials (ERPs), Cognitive control, Neural adaptation, Neural modification

## Abstract

**Background:**

Individuals with lower-limb amputations are highly dependent upon prostheses to perform daily activities and adapt to environmental changes. Transtibial prosthesis (TTP) users in particular, experience greater challenges in motor control and demonstrate impaired cognitive functions, when compared to able-bodied persons. The identification of neural mechanisms underlying adaptation or compensation may contribute to the development and expansion of rehabilitation strategies.

**Objective:**

To examine neuroplasticity changes in transtibial amputees by analyzing event-related potentials (ERPs) obtained from Electroencephalogram (EEG) during Go/No-Go tasks to assess cognitive control and neural adaptations.

**Methods:**

Twenty-eight TTP users and twenty-eight able-bodied persons were recruited. EEG was recorded in eyes-open resting states, and ERPs during a Go/No-go task.

**Results:**

Our findings demonstrate that, during the resting-state, the TTP users group exhibited no significant differences in brain activity across regions. However, during Go/No-go task, an increase of N2 amplitude was observed, and significant reduction in the amplitude of P3 amplitude was noted when compared to able-bodied group.

**Conclusion:**

These findings demonstrated neural modifications by individuals with transtibial amputation, particularly in relation to inhibitory control, which is essential for effective attentional control. Deficits in inhibitory control may interfere with decision-making processes, thereby impairing the execution of daily activities that require sustained attention and cognitive flexibility. Based on these findings of neural adaptions, it may be necessary to consider targeted interventions aimed at enhancing cognitive control and incorporating specific cortical training strategies for TTP users.

## Introduction

Many people are living with limb amputations and limb differences worldwide. These individuals depend on prostheses to engage in daily activities and often face difficulty environmental challenged which require flexibility and behavior adaptations. Advances in prosthetic development have improved the quality of life of prostheses users. However, there are limitations associated with the use of prostheses. These include compensatory movements, reduced gait performance and increases in fatigue. There has been considerable effort to mitigate the aforementioned issues and optimizing function through advanced technology, biomedical engineering, and innovative rehabilitation methods [1].

One major concern for unilateral trans-tibial prosthesis users is greater difficulties in movement control when compared to able-bodied persons, particularly in motor control scenarios involving motor planning, decision-making or inhibitory responses [2]. Previous reports show that amputees have impaired psychomotor function, such as perceptual and executive processing. The functional impairments observed in amputees may be partially compensated or adapted through open-skill development, particularly in dynamic and unpredictable environments [3, 4]. In addition, Yuanyuan L. et al., (2016) demonstrated resting-state network reorganization following upper limb amputation [5]. This finding warrants further investigation into whether comparable reorganization occurs in lower limb amputees. While principles of neural plasticity, cortical homunculus organization, and motor planning suggest a potential for similar effects in lower limbs, the precise nature and extend of such reorganization remain unclear. In this study, brain reorganization is considered a manifestation of neuroplasticity, reflecting the ongoing neural modification that allow the brain to reorganize itself by forming new neural connections in response to experience [6]. Although neuroplasticity has not been directly measured, its presence can be inferred from the observed responses and corresponding changes in brain activity, which suggest functional reorganization within the brain areas and associated process [7].

Furthermore, amputees have been shown to exhibit cognitive impairments such as reduced reasoning, information processing, attention, memory, language/naming, and visuospatial function [8]. Amputation may alter the organization of neural pathways, affecting how individuals adapt to their environment, particular in terms of inhibition control. The consequences of amputation may elicit changes in neural pathways [9], decreased proprioception [10], phantom sensations [11], and lack of embodiment of the prosthetic limb [12]. The interplay of these cognitive burdens after limb loss over a prolonged period may necessitate more attention and concentration required to complete tasks, and frustration arising during prostheses use [13].

Considering the crucial role that neuroplasticity plays in cognitive control, implementation of neuroplasticity-focused strategies for neuroprosthetic development and rehabilitation programs could be advantageous for individuals with amputations. A comprehensively understanding and applying the principles of neuroplasticity can enhance intuitive and functional prosthetic control, particular for activities of daily living, allowing individuals to regain independence and improve their quality of life. Targeted training approaches (e.g., tasks-specific training, progressive difficulty paradigms, virtual reality-based interventions) have been shown to promote neuroplastic adaptation [14, 15]. Furthermore, brain computer interfaces (BCIs) and neuromodulation techniques including transcranial magnetic stimulation (TMS) and transcranial direct current stimulation (tDCS) can further facilitate the learning and adaptation of neural process, thereby optimizing motor and cognitive rehabilitation [16, 17]. Therefore, elucidating underlying inhibitory mechanisms in people with transtibial prostheses would guide the development of advanced rehabilitation and neuroprosthetic technologies. Consequently, mitigating loss of efficient cognitive control in amputees who have experienced prolonged periods of limb loss is a critical challenge in rehabilitation approaches. Therefore, insight in amputee neuroplasticity strategies may help guide treatment during prosthesis or wheelchair use [18], functional mobility, and locomotor rehabilitation [19].

Electroencephalogram (EEG), known for a its high temporal resolution, has been utilized to investigate the neural signatures associated with motor learning through brain oscillations. The magnitude and frequency of beta band oscillation (13–30 Hz) has revealed the main neural signatures of voluntary movement and motor learning which are related to GABAergic neural activity [20–22]. In particular, the motor cortex preceding voluntary movement reveals negative potentials in a type of Event-related potentials (ERPs) with low frequency [23]. This has been previously identified as an important marker of motor inhibitory processes at the negative peak around 250–300 milliseconds [24, 25]. It is these anticipatory movements that reflect the cortical processes in planning and preparation. In experimental scenarios aimed at investigating cognitive control, a widely employed Go/No-Go, stop-signal and continuous performance task are used to examine these cognitive processes; attention, decision-making, working memory and inhibitory control [26–28]. Warranting these methods for assessing neural modification in cognitive and motor tasks [29, 30].

The purpose of this study was to investigate neural modification in individuals with transtibial amputation using EEG extracted ERPs. Comparisons of neural activity between TTP users and able-bodied persons were assessed during a resting-state brain activity and the Go/No-go tasks via visual stimuli of both groups. It is was hypothesized that participants with amputation would exhibit neural modifications during specific brain activities.

## Materials and methods

### Participants

The study was conducted in alignment with the principles outlined in the Declaration of Helsinki and received approval from the Institutional Review Board of the Faculty of Medicine Siriraj Hospital, Mahidol University (SIRB Protocol No. 959/2564 (IRB1), approval date: 29 August 2022). A total of fifty-six participants were included in the study, each of whom provided informed consent prior to data collection. There were two groups which consisted of twenty-eight transtibial prosthesis (TTP) users (24 males and 4 females, 52.8 ± 13.5 y, 166.2 ± 7.2 cm, 64.8 ± 10.0 kg, mean ± SD), and twenty-eight able-bodied persons (14 males and 14 females, 40.8 ± 12.7 y, 163.5 ± 10.8 cm, 67.8 ± 14.4 kg, mean ± SD). Inclusion criteria of transtibial prosthesis wearers was: (1) unilateral transtibial amputation (> 6 months) with medium to long residual limb length, (2) ability to walk for extended periods, and amputee K-Level functional levels 1–4, (3) normal range of motion (ROM) in all joints of the lower limb, (4) lower limb muscle strength grade 4–5 according to the Oxford scale, and (5) ability to comply with directions and comprehend study procedures. All participants were in good health, could walk without a cane or other aid, and had a normal range of motion in lower limb joints. Participants with current or recent (within the past 3 month) episodes of phantom limb pain that interfere with their ability to participate in the study were excluded. The prosthetist checked each prosthesis to ensure optimal functioning, and that participants were capable of performing study procedures. Seventeen TTP users wore prostheses on their right leg, while eleven wore prostheses on their left leg. Among them, 24 individuals exhibited right-leg dominance, whereas 4 demonstrated left-leg dominance. All able-bodied participants demonstrated right-side dominance.

### Experimental setup and design

**Fig. 1:**
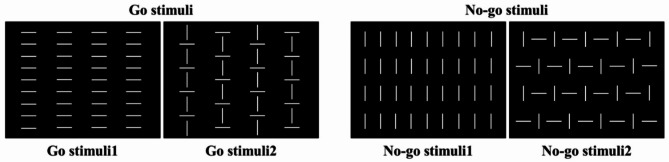
The four insets show the stimuli used in the experiment for Go (left) and No-go (right) tasks

Both groups completed the same two tasks: (1) EEG recordings during resting-state, and (2) ERPs recordings during Go/No-go tasks in response to visual stimuli [25]. In the resting-state, participants were seated in a chair in a relaxed position at the laboratory room. They were instructed not to move; data was recorded for 5 min while their eyes remained open in a rested-state. In Go/No-go tasks, two configurations were defined as targets (Go stimuli) and two nontargets (No-go stimuli) as show in Fig. 1. The participants were instructed to press a button with their dominant hand as quickly as possible when a target appeared on the screen (Go stimuli; 0.5), and withheld the response when non-target appeared (No-go stimuli; 0.5). There were three trails of the Go/No-go tasks, each trial utilizing a total of 80 pictures which included Go stimuli1, Go stimuli2, No-go stimuli1, No-go stimuli2 (20 pictures for each type). The picture stimuli were presented in random order during each trial, and each trial lasted 2-minutes. Participants were given a 5-minute rest between trials, and the duration of the two experiments was 30 min. Figure 1, illustrates the squared configurations made of vertical and horizontal bars subtending 4 × 4◦ and were presented for 200 milliseconds on a dark grey background. The four configurations were displayed randomly with an equal probability (0.25); onset asynchrony varied from 1 to 2 s.

### Sensor outcome measures: EEG recording and analysis

An EEG recording system (eego™mylab ANT Neuro, USA) was used and was comprised of 32-channels; Fp_1_, Fp_2_, Fp_z_, F_3_, F_4_, F_7_, F_8_, F_z_, FC_1_, FC_2_, FC_5_, FC_6_, C_3_, C_4_, C_z_, T_7_, T_8_, CP_1_, CP_2_, CP_5_, CP_6_, P_3_, P_4_, P_7_, P_8_, P_z_, PO_z_, O_1_, O_2_, O_z_, M_1_, and M_2_, along with a reference electrode (REF = CP_z_) and ground electrode (GND). The electrode impedances were maintained below 20 kΩ, as recommended in prior scholarship [31]. An online filter was set to bandpass filter between 0.3 and 30 Hz. The sampling rate was set at 512 Hz and notch filter was set at 50 Hz. The absolute power spectrum of the respective frequency bands derived by Fast Fourier Transformation (FFT) was expressed as follows: Delta (0.3–4 Hz), Theta (4.5–8 Hz), Alpha (8.5–13 Hz) and beta (13.5–30 Hz) wave ranges. The ERPs recordings, and peak amplitudes and latencies of the N2 and P3 waves were measured over the midline occipital (O_z_), parieto-occipital (PO_z_), midline parietal (P_z_), midline central (C_z_), and midline frontal (F_z_) electrode sites. The peak amplitudes (measured with respect to 100 millisecond pre-stimulus baseline) and latencies of major ERPs component were calculated for each participant in the following time window: N2 within 200–300 ms [32], and P3 within 250–500 ms [25]. The peak amplitude (μV) was defined as the voltage difference between the baseline and the negative- and positive-going peak of the ERPs waveform after stimulus presentation. The peak latency (ms) was defined as the time from stimulus onset to the point of the maximum negative and positive amplitude of the N2 and P3 waves respectively. In this study, we conducted two types of ERPs analyses: simple reaction analysis and discriminative reaction analysis. The simple reaction analysis categorized stimuli into two types: Go stimuli (requiring a response) and No-go stimuli (requiring inhibition). In contrast, the discriminative reaction analysis further divided the stimuli into four categories: Go stimuli1, Go stimuli2, No-go stimuli1, and No-go stimuli2, allowing for an examination of the neural mechanisms underlying stimulus discrimination and response selection. In the analysis, the correct Go responses (Go1, and Go2 stimuli) were used to compute the average amplitude and latency of ERPs.

### Statistical analysis

All statistical tests were performed in Jamovi, and data visualization within the R statistical framework (version 4.0, R Foundation for Statistical Computing) and ASA software. Three metrics were of interest in this study; EEG during resting-state, ERPs during Go/No-go tasks, and physical response when participants pushed down with their hand during Go/No-go tasks. To assess the normality of the data, Shapiro-Wilk tests were initially applied, revealing an abnormal distribution of the data. Therefore, a generalized linear mixed modelling (GLMM) was used to report the linear predictor between EEG during resting-state in brain areas (random), and groups (fixed) effects. The Kruskal-Wallis one-way ANOVA was used to compare groups with respect to peak amplitude and latency of N2 and P3 of the ERPs recording, response time, and the number of correct responses. Following a significant Kruskal-Wallis’s test result, Dwass-Steel-Critchlow-Fligner (DSCF) pairwise comparisons were performed to identify specific group differences while controlling for multiple comparisons. Only the results demonstrating statistical significance or addressing our hypothesis are presented in this study. The significance level was set at *ρ* < 0.05.

## Results

The results in this study demonstrate findings related to neural modification of transtibial prosthesis (TTP) users. EEG data during resting-state were analyzed according to four frequency bands; delta, theta, alpha, and beta band. ERPs data during Go/No-go tasks to visual stimuli were analyzed in peak amplitudes and latencies of N2 and P3. Physical responses were analyzed for response time, and the number of correct responses.

### EEG during resting-state

**Fig. 2:**
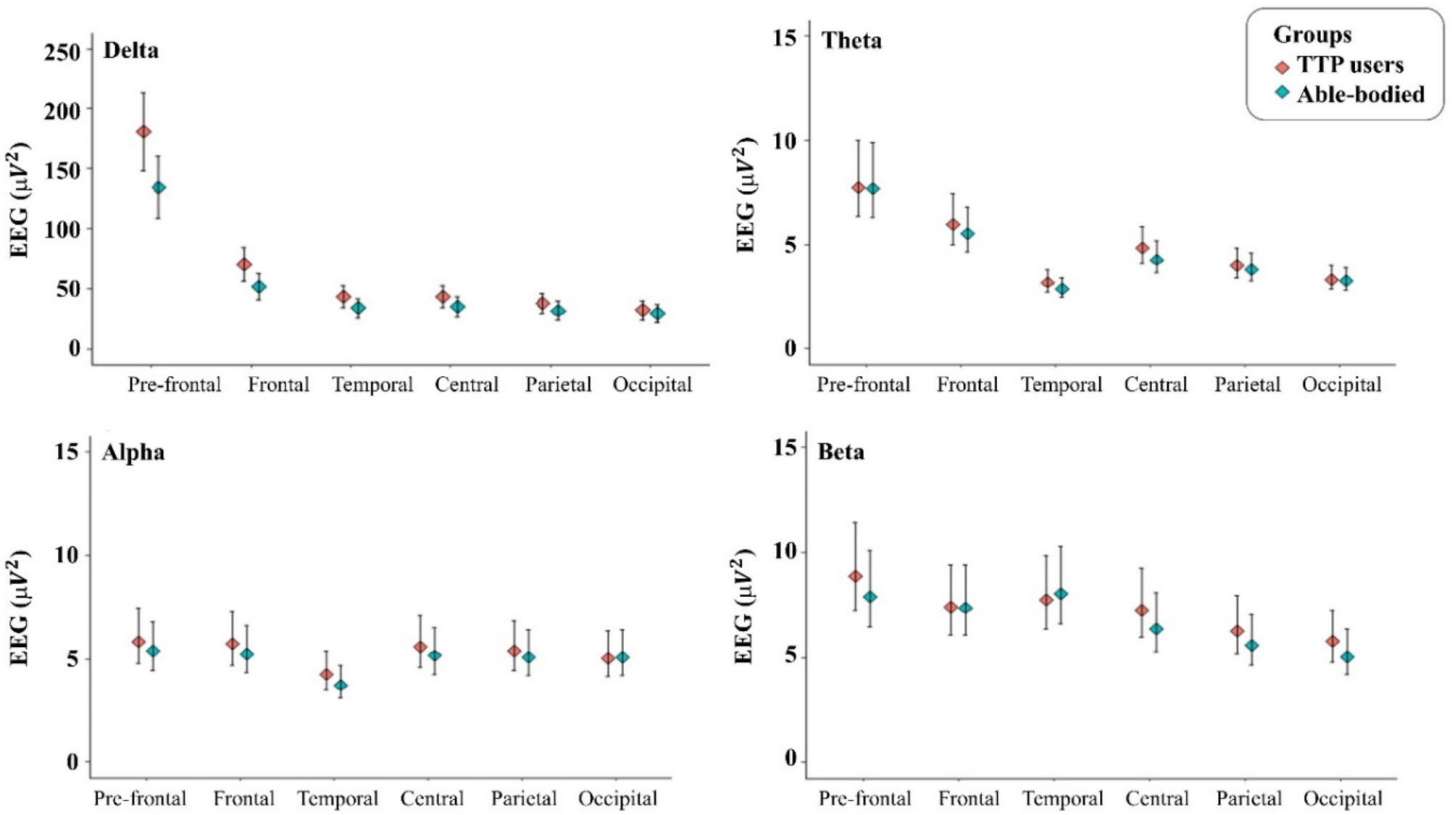
The mean values and 95% CI of power spectral density for EEG activity during the resting-state condition

There was no a significant difference between TTP users and able-bodied persons in all EEG frequency bands. The mean values and 95% confidence intervals (95% CI) of power spectral density for EEG activity during the resting-state condition are shown in Fig. 2.

### ERPs during Go/No-go task to visual stimuli

**Fig. 3:**
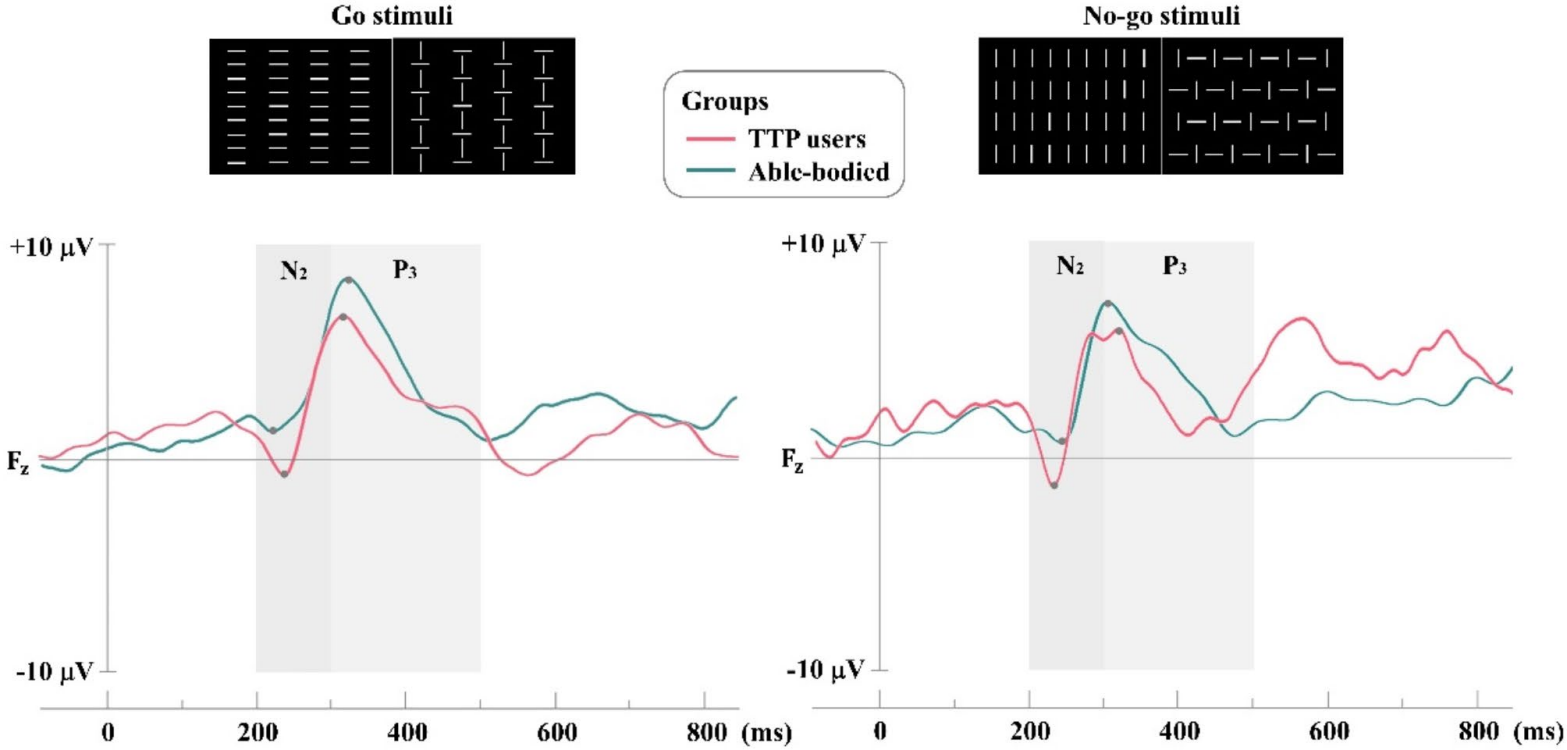
The average of N2 and P3 amplitudes for simple reaction analysis

**Fig. 4:**
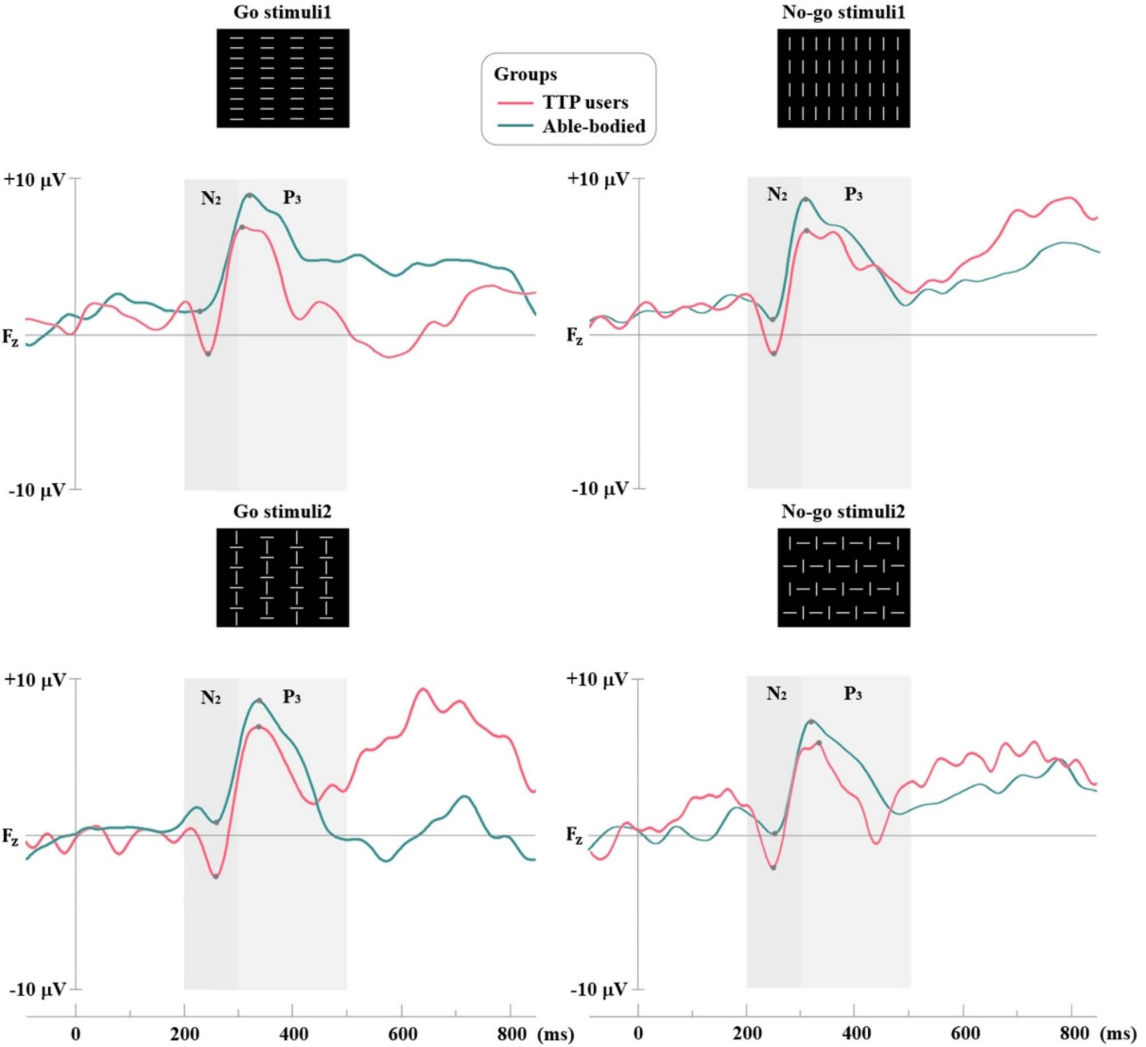
The average of N2 and P3 amplitudes for discriminative reaction analysis

Tables 1 and 2 present the mean values, 95% confidence intervals (lower and upper bounds), standard deviation (SD) and *ρ*-values for the amplitude differences in the N2 and P3 components at the F_z_ electrode position across all conditions, respectively. The amplitude of N2 differed between groups in both simple and discriminative reaction analyses. In simple reaction analysis, there were significant differences between groups for both Go stimuli (*ρ* = 0.007, DSCF-adjusted) and No-go stimuli (*ρ* = 0.002, DSCF-adjusted) targets. The N2 amplitude at the F_z_ position was observed in both TTP users (Go: average = -0.940 μV, No-go: average = -1.370 μV), and able-bodied (Go: average = 1.550 μV, No-go: average = 1.020 μV) as shown in Table 1; Fig. 3. In discriminative reaction analysis, there were significant differences between groups for Go stimuli1 (*ρ* = 0.007, DSCF-adjusted), Go stimuli2 (*ρ* = 0.013, DSCF-adjusted), No-go stimuli1 (*ρ* = 0.001, DSCF-adjusted), and No-go stimuli2 (*ρ* = 0.007, DSCF-adjusted) targets. The N2 amplitude at the F_z_ position in TTP users was recorded as: Go1: average = -1.300 μV, Go2: average = -2.490 μV, No-go1: average = -1.300 μV, and No-go2: average = -2.060 μV. In contrast, the positive peak in able-bodied was as follows: Go1: average = 1.810 μV, Go2: average = 0.380 μV, No-go1: average = 1.260 μV, and No-go2: average = 0.091 μV as shown in Table 1; Fig. 4.

**Table 1:** Descriptive statistics for the amplitude differences in the N2 component at the F_z_ electrode position across all conditions

Analysis	Stimuli	ERP measures					Descriptive statistics					
			Mean			95% CI LB		95% CI UB		SD		*ρ* (DSCF-adjusted)
			TTP users	Able-bodied		TTP users	Able-bodied	TTP users	Able-bodied	TTP users	Able-bodied	
Simple	Go	Amp	-0.940	1.550		-2.170	0.336	0.294	2.770	3.180	3.140	0.007*
		Lat	-	-		-	-	-	-	-	-	-
	No-go	Amp	-1.370	1.020		-2.350	0.014	-0.391	2.030	2.530	2.600	0.002*
		Lat	-	-		-	-	-	-	-	-	-
Discriminative	Go1	Amp	-1.300	1.810		-2.830	0.386	0.245	3.220	3.970	3.660	0.007*
		Lat	-	-		-	-	-	-	-	-	-
	Go2	Amp	-2.490	0.383		-4.340	-0.864	-0.643	1.630	4.770	3.220	0.013*
		Lat	-	-		-	-	-	-	-	-	-
	No-go1	Amp	-1.300	1.260		-2.210	-0.016	-0.397	2.540	2.330	3.300	0.001*
		Lat	-	-		-	-	-	-	-	-	-
	No-go2	Amp	-2.060	0.091		-3.170	-0.804	-0.947	0.985	2.860	2.310	0.007*
		Lat	-	-		-	-	-	-	-	-	-

* Significance level *ρ* < 0.05

Abbreviations: Amp = amplitude (μV), Lat = latency (ms), TTP users = transtibial prosthesis users

In addition, the amplitude of P3 differed between groups only No-go task in both simple and discriminative reaction analyses as shown in Fig. 3, and 4 respectively. There were significant differences between groups in simple reaction analysis (No-go stimuli: *ρ* = 0.044, DSCF-adjusted), and discriminative reaction analysis (No-go stimuli1: *ρ* = 0.020, DSCF-adjusted, and No-go stimuli2: *ρ* = 0.049, DSCF-adjusted). The P3 amplitude at the F_z_ position of able-bodied was greater than TTP users in all No-go stimuli tasks. Maximum P3 amplitude was observed in No-go stimuli (TTP users: 5.830 μV and able-bodied: 7.160 μV), No-go stimuli1 (TTP users: 6.550 μV and able-bodied: 8.500 μV), and No-go stimuli2 (TTP users: 6.000 μV and able-bodied: 7.540 μV) as shown in Table 2. However, no significant difference was found in the latency of N2 and P3 between groups in both simple and discriminative reaction analyses.

**Table 2:** Descriptive statistics for the amplitude differences in the P3 component at the F_z_ electrode position across all conditions

Analysis	Stimuli	ERP measures					Descriptive statistics					
			Mean			95% CI LB		95% CI UB		SD		*ρ* (DSCF-adjusted)
			TTP users	Able-bodied		TTP users	Able-bodied	TTP users	Able-bodied	TTP users	Able-bodied	
Simple	Go	Amp	-	-		-	-	-	-	-	-	-
		Lat	-	-		-	-	-	-	-	-	-
	No-go	Amp	5.830	7.160		4.770	6.080	6.880	8.240	2.720	2.780	0.044*
		Lat	-	-		-	-	-	-	-	-	-
Discriminative	Go1	Amp	-	-		-	-	-	-	-	-	-
		Lat	-	-		-	-	-	-	-	-	-
	Go2	Amp	-	-		-	-	-	-	-	-	-
		Lat	-	-		-	-	-	-	-	-	-
	No-go1	Amp	6.550	8.500		5.390	7.170	7.700	9.840	2.990	3.440	0.020*
		Lat	-	-		-	-	-	-	-	-	-
	No-go2	Amp	6.000	7.540		5.030	6.420	6.970	8.650	2.500	2.870	0.049*
		Lat	-	-		-	-	-	-	-	-	-

* Significance level *ρ* < 0.05

Abbreviations: Amp = amplitude (μV), Lat = latency (ms), TTP users = transtibial prosthesis users

### Physical responses during Go/No-go task to visual stimuli

Response time results demonstrated no significant differences between TTP users and able-bodied persons in both analyses. However, the number of correct responses revealed significant differences in simple reaction analysis (*ρ* = 0.017), and descriptive reaction analysis at Go2 (*ρ* = 0.031). Descriptive statistics of response time and number of correct responses including the mean values and 95% confidence intervals (lower and upper bounds) are shown in Table 3.

**Table 3:** Descriptive statistics of number of correct responses (times) data including the mean values, SD, and 95% confidence intervals

Analysis	Stimuli	Physical measures					Descriptive statistics					
			Mean			95% CI LB		95% CI UB		SD		*ρ* (DSCF-adjusted)
			TTP users	Able-bodied		TTP users	Able-bodied	TTP users	Able-bodied	TTP users	Able-bodied	
Simple	Go	RT	-	-		-	-	-	-	-	-	-
		NCR	15.60	17.60		14.20	16.40	17.00	18.70	3.790	3.050	0.017*
Discrimi-native	Go1	RT	-	-		-	-	-	-	-	-	-
		NCR	-	-		-	-	-	-	-	-	-
	Go2	RT	-	-		-	-	-	-	-	-	-
		NCR	14.70	17.00		12.90	15.60	16.50	18.40	4.830	3.780	0.031*

* Significance level *ρ* < 0.05

Abbreviations: RT = responses time (ms), NCR = the number of correct responses (times)

## Discussion

The present study examined whether transtibial prosthesis (TTP) users evidenced neural modifications in brain activity during resting-state, and a Go/No-go task to visual stimuli. We hypothesized that participants with amputation would exhibit neuroplasticity within specific brain activity. Our main findings between the TTP user and able-bodied groups were as follows: (1) during resting-state, no significant differences were observed between groups across brain regions (2) in the Go/No-go tasks to visual stimuli, the TTP users group exhibited increased N2 amplitude in their response and a significant reduction of P3 amplitude, (3) the TTP users group demonstrated a lower number of correct responses during Go/No-go tasks.

Prior studies have reported brain reorganization following amputation, including structural changes such as gray matter reduction within the hand representation in upper limb amputees [33], as well as alterations in white matter within the corpus callosum in lower limb amputees [34]. Likewise, assessment of the neural efficiency in communication and integration across brain regions has been conducted to demonstrate neural activity changes among people with upper [5] and lower limb amputation [35, 36]. The present study employed measures of resting-state neural activity, which are proving highly useful at reflecting the intrinsic properties of the brain network including neuroanatomical structure, local neuronal dynamics, and functional potential of the brain [37]. However, no differences were found in any EEG frequency bands during resting state between TTP users and able-bodied. As expected, neural activity measurement using EEG did not contribute to resting state response. This could indicate the normality of spontaneous neural fluctuation in both TTP user and able-bodied groups [38]. The absence of statistically significant differences in resting state might be attributed to the amputation characteristics, including an individual’s familiarity with their prosthetic device, the rehabilitation protocol, and the lack of challenges or activities related to neural and cognitive functions [39]. These factors could potentially mitigate variability in resting state measures, thus influencing the observed outcomes.

In addition, our study employed Go/No-go paradigm to assess the N2 and P3 ERPs component, and to observe the neural basis of motor response execution and inhibition. Though, the resting state did not show any significant different, in evaluations requiring cognitive involvement such as Go/No-go task, the results were contrary. Different ERPs brainwaves of TTP user and able-bodied groups are shown in Figs. 3 and 4 (simple and discriminative reaction analysis). The TTP user group demonstrated a significant increase in negative peak at 200 ms (N2) on the frontal site (F_z_) compared to able-bodied persons for both Go and No-go stimuli, as observed in both simple and discriminative reaction analysis. The increased N2 amplitude at F_z_ in the TTP user group indicate a distinct pattern in the cognitive control mechanisms of TTP users brain, particularly in aspects related to inhibitory and selective attention [40]. Response inhibition pertains to a suppressed action that is contextually inappropriate and may disrupt behavioral control, particular in TTP users. Additionally, the presence of N2 in stimulation response reflects the brain’s effort to monitor and manage the mismatch detection between a prepared motor response and requirement to inhibit it [41]. This discrepancy suggests that while baseline neural activity may be similar across groups, tasks demanding cognitive engagement can reveal differences in brain function and processing.

Additionally, both simple and discriminative reaction analysis revealed a significant reduction in P3 amplitude in the TTP user group during No-go trails, while no significant differences were observed between groups during Go trials. The observed P3 wave elicited by Go/No-go stimuli reflects cognitive process associated to attention, stimulus evaluation, response inhibition and decision making [42]. Remarkably, the reduction in P3 amplitude in TTP user group may indicate a diminished allocation of attention and cognitive resources toward inhibiting inappropriate or irrelevant actions, in contrast to the able-bodied group [43]. It is the demonstration of a neural response indicating the engagement of cognitive resources necessary for evaluating the relevant stimuli and inhibiting inappropriate response, which are critical for effective cognitive control [44]. This finding may suggest that individuals in the TTP user group could have experience challenges in inhibitory control, potentially affecting their ability to focus on relevant stimuli and manage responses effectively. This aligns with the analysis of number of correct responses and response time during go trials. Our results indicate that the TTP user group exhibited lower correct responses compared to able-bodied group, while their response times were comparable. This suggests that, in terms of the physical reaction to stimuli, the TTP user group responded as quick as the able-bodied participants. However, when considering accuracy, the TTP user group appeared to have a reduced capability for selecting the correct response. This could lead to the presumption that TTP users may have impaired inhibitory control and attentional processing compared to the able-bodied group. This finding is consistent with our neural data, which demonstrate alterations in brainwave activity, including the decreased amplitude of P2 and increased N2 amplitude in Go/No-go response. Our findings are consistent with cognitive response potentially related to the TTP. These neurophysiological changes align with the concept of neural plasticity, reflecting the brain’s adaptative mechanism in response to the use of prosthetic use [7].

These results tie in well with the assumption that people with transtibial amputation undergo neural modifications within the mechanisms responsible for adapting their behavior to the environment. The observed differences in cognitive function at frontal midline (Fz) brain region among TTP users suggest that the cortical motor planning process may also play a role [45]. This involvement is evident as motor planning process contribute to the regulation and execution responses in Go/No-Go tasks, which require cognitive control and response inhibition [46]. These changes may be influenced by neuroplastic adaptation within the corticospinal tract, potentially leading to altered transmission of motor response signals to the prosthetic limb [47]. Thus, motor response selection and inhibition processes are crucial. These neural modifications or compensations in the TTP user group may interfere with successful execution of everyday tasks, such as stopping at traffic lights, preventing interruptive or impulsive verbal behavior, and patiently waiting in line. Moreover, response inhibition is considered crucial for attentional control, as the ability to inhibit responses to distracting stimuli is essential for maintaining focus and sustaining task-oriented behavior [40]. Understanding how the brain adapts following amputation is essential for informing rehabilitation strategies and prosthetic design. Insights into this neural modification can be applied to optimize rehabilitation techniques, enhance the suitability of lower limb amputees for prosthetic or wheelchair use, and improve functional mobility and locomotor relearning. By tailoring rehabilitation protocols to account for brain plasticity, clinicians can more effectively support the reintegration of motor and cognitive functions, ensuring a more efficient and personalized approach to improving quality of life for transtibial prosthesis users. Furthermore, the findings regarding neural adaption may necessitate specific cortical training intervention for amputees, including techniques such as motor imagery, motor planning exercise, the utilization of virtual reality [48], and brain-computer interface technology [49]. These approaches can facilitate the reorganization of cortical areas, potentially enhancing motor control and functional relearning in individual who have undergone amputation.

One of the limitations of this study is the absence of recorded response times and number of corrections during unsuccessful inhibition in the No-go trails. These variables could provide critical insights into the differential engagement of brain regions commonly involved in inhibitory control. It could illustrate how neural mechanisms contribute to performance in inhibitory tasks, offering insights into significant processes related to error monitoring and response corrections, which plays a significant role in cognitive control and adaptive behavior [50]. Future research should consider using alternative tasks that more closely simulate real-life and scenarios involving prosthetic use, such as object manipulation or activity daily living, to more comprehensively asses the neural adaptation associated with prosthetic limb integration. This would allow for more accurate evaluations of how neural processing adaptations are influenced under conditions that better reflect real world environments. Although our sample did not include individuals with significant phantom limb pain, future research could focus on investigating this specific condition to determine whether distinct neural adaptation patterns associated with prosthetic use in the presences of phantom limb pain. Additionally, studies should explore the differential effects of left and right limb amputations on neural adaptation. This approach would allow for a deeper understanding of hemispheric-specific neural changes that occur as a result of amputation, further enhancing the development of tailored rehabilitation strategies.

## Conclusion

In this study, no significant differences were observed in resting-state EEG data between transtibial prosthesis (TTP) users and able-bodied. However, neural modifications were evident in TTP users during cognitive tasks, as indicated by the presence of N2 and reduction in P3 amplitude during Go/No-Go tasks. These findings suggest a reorganization of cognitive processing efficiency, particularly in inhibitory control and attentional modulation. Inhibition plays a vital role in filtering out distraction stimuli to maintain focus and support task-oriented behavior. Impairments in inhibitory control may interfere with decision-making processes, potentially compromising the execution of daily activities that require sustained attention and cognitive flexibility. Such neural modifications could therefore have significant implications for functional capabilities and quality of life of TTP users.

## Data Availability

No datasets were generated or analysed during the current study.

## Abbreviations

Amp: Amplitude (μV)
DSCF: Dwass-Steel-Critchlow-Fligner
EEG: Electroencephalogram
ERPs: Event-Related Potentials
FFT: Fast Fourier Transformation
GLMM: Generalized Linear Mixed Modelling
GND: Ground Electrode
Lat: Latency (ms)
N2: Negative peak within 200–300 ms
NCR: Number of Correct Responses (times)
P3: Positive peak within 250–500 ms
REF: Reference Electrode
ROM: Range of Motion
RT: Responses Time (ms)
TTP: Transtibial Prosthesis
